# Differential expression of connective tissue growth factor and hepatocyte growth factor in the vitreous of patients with high myopia versus vitreomacular interface disease

**DOI:** 10.1186/s12886-019-1041-1

**Published:** 2019-01-21

**Authors:** Xinyi Ding, Rong Zhang, Shujie Zhang, Hong Zhuang, Gezhi Xu

**Affiliations:** 1 Department of Ophthalmology, Eye and ENT Hospital of Fudan University, 83 Fenyang Road, Shanghai, 200031 China; 2 Eye Institute, Eye and ENT Hospital of Fudan University, Shanghai, China; 3 Key Laboratory of Visual Impairment and Restoration of Shanghai and Key Laboratory of Myopia of State Health Ministry, Fudan University, Shanghai, China

**Keywords:** Connective tissue growth factor, Hepatocyte growth factor, High myopia, Vitreomacular interface disease

## Abstract

**Background:**

To determine the levels of connective tissue growth factor (CTGF) and hepatocyte growth factor (HGF) in the vitreous of patients with high myopia, in comparison with those with a vitreomacular interface disease (VMID).

**Methods:**

Patients with either high myopia (high myopia group) or a VMID (VMID group) were included in this study. Each of the two groups were further subdivided into two subgroups: group A (high myopia with macular hole), group B (high myopia with macular retinoschisis), group C (idiopathic macular hole), and group D (idiopathic epiretinal membrane). Vitreal specimens were collected during vitrectomy, and enzyme-linked immunosorbent assay was used to quantitatively measure the CTGF and HGF levels in the vitreous.

**Results:**

The average axial length was markedly longer in the high myopia group than in the VMID group. The vitreal CTGF level was significantly higher in the high myopia group than in the VMID group. Subgroup analysis revealed significantly higher vitreal CTGF in group A than in the other three subgroups. The vitreal HGF level was not significantly different between the high myopia and VMID groups, but was significantly higher in group D than in group C in the subgroup analysis. Correlation analysis showed that the vitreal CTGF level was positively correlated with the axial length.

**Conclusions:**

The vitreal CTGF level is elevated in highly myopic eyes and may be related to the pathogenesis of high myopia, whereas increased expression of HGF may be involved in the development of idiopathic epiretinal membrane.

## Background

Myopia is prevalent worldwide, particularly in East Asian countries such as China [1–3]. According to an epidemiological study, the incidence of high myopia is increasing.^2^ High myopia is characterized by progressive elongation of the eyeball, scleral thinning, and formation of posterior staphyloma. The posterior segment complications in high myopia, which include retinal detachment, choroidal neovascularization, macular retinoschisis, and macular hole, are leading causes of irreversible vision loss [4, 5].

Myopia is caused by remodeling of the scleral extracellular matrix. Animal studies indicate that cytokines and proteinases are involved in the scleral remodeling processes during the development of myopia. Previous studies have focused mainly on transforming growth factor (TGF)-β and matrix metalloproteinases (MMPs) [6–8]. We previously measured TGF-β2 and MMP-2 levels in the vitreous of patients with high myopia and found that the MMP-2 level was elevated and strongly correlated with the TGF-β2 level in high myopia [9].

Other cytokines may also be involved in the development of myopia. Connective tissue growth factor (CTGF) is expressed by fibroblasts in various tissues and is involved in the synthesis of the extracellular matrix [10, 11]. Hepatocyte growth factor (HGF) upregulates the expression of MMP-2, which results in degradation of extracellular matrix [12]. Expression of HGF was increased in the scleral tissue of a myopic animal model [13]. However, the expression of CTGF and HGF has not been evaluated in human myopic eyes.

Therefore, we investigated the changes in CTGF and HGF levels in the vitreous of patients with high myopia. As a comparison, we also evaluated these vitreal cytokines in patients with the vitreomacular interface diseases (VMIDs) of idiopathic macular hole and idiopathic epiretinal membrane.

## Methods

### Subjects

We performed an observational study of patients with either high myopia (high myopia group) or a VMID (VMID group) who underwent vitrectomy surgery at the Eye and ENT Hospital of Fudan University, China. The present study was conducted in accordance with the principles of the Declaration of Helsinki and was approved by the Ethics Committee of the Eye and ENT Hospital of Fudan University.

High myopia was defined as a spherical equivalent ≤ − 6.0 diopter and axial length ≥ 26 mm. A total of 33 eyes of 33 patients with high myopia who received vitrectomy for macular hole or macular retinoschisis were enrolled in this study. Among these eyes, 19 had macular hole and 14 had macular retinoschisis. Exclusion criteria included those high myopic patients with choroidal neovascularization, proliferative vitreoretinopathy, and any history of laser treatment or ocular surgery.

As a comparison, patients with VMID were also enrolled in this study. A total of 33 eyes of 33 patients with VMID were evaluated: 18 eyes with idiopathic macular hole and 15 with idiopathic epiretinal membrane. None of these eyes had high myopia or any history of ocular trauma or intraocular inflammation.

All patients received a comprehensive ocular examination, including an ophthalmoscopic examination, A- and B-scan ultrasonography, and optical coherence tomography. Axial length was assessed by A-scan ultrasonography and posterior staphyloma by B-scan ultrasonography in each patient with high myopia. The diagnoses of macular retinoschisis, macular hole, and epiretinal membrane were made using optical coherence tomography.

Samples of undiluted vitreous fluid (300–500 μl) were collected in sterile tubes at the time of vitrectomy before intraocular infusion and then immediately stored at − 80 °C until assayed.

### Measurement of CTGF and HGF levels

Enzyme-linked immunosorbent assay kits were used to measure the concentration of CTGF (Assay Biotech, Losangeles, USA) and HGF (R&D Systems, Minneapolis, USA) according to each manufacturer’s protocol. In brief, the samples were added to the wells of a 96-well plate coated with a monoclonal antibody and incubated for 2 h, after which the plate was washed and an enzyme-labeled antibody added. After further incubation, the plate was washed again, and the substrate was added. Color development was terminated using the stop solution provided in the kits, and the optical density was read at 450 nm and 630 nm using a multimode microplate reader (Synergy H1, BioTek Instruments, USA). The 450 nm readings corrected by the 630 nm readings were used for evaluation. A standard curve was generated by measuring the optical densities of serially diluted protein solutions of known concentrations. The concentrations of CTGF and HGF in the vitreous samples were calculated based on the standard curve.

### Statistical analysis

Statistical analysis was performed using Stata 11.0 statistical software (Stata Corporation, College Station, TX, USA). Differences between the high myopia and VMID groups were analyzed using the Wilcoxon rank-sum test. Each of the two groups (high myopia and VMID groups) were further subdivided into two subgroups: group A (high myopia with macular hole), group B (high myopia with macular retinoschisis), group C (idiopathic macular hole), and group D (idiopathic epiretinal membrane). Differences among the four subgroups were analyzed using the Kruskal–Wallis test. The correlations between two parameters were analyzed using Spearman’s rank correlation coefficient. A two-tailed *P* value < 0.05 was considered statistically significant.

## Results

### Patient characteristics

The high myopia group included 24 females and 9 males, and the VMID group included 25 females and 8 males. Thus, both groups comprised mainly females, and there was no difference in sex distribution between the two groups. The average patient age was significantly lower in the high myopia group (55.3 ± 9.9 years) than in the VMID group (61.8 ± 5.6 years; *P* < 0.01). The average axial length was markedly longer in the high myopia group (28.3 ± 1.7 mm) than in the VMID group (23.2 ± 1.0 mm, *P* < 0.0001). The subgroup analysis of axial length showed no difference between groups A and B or between groups C and D. The patient characteristics and vitreal cytokine concentrations in the four subgroups are listed in Table 1.

**Table 1:** Patient characteristics and vitreal cytokine concentrations in four subgroups

Parameter	Group A	Group B	Group C	Group D
Gender (female/male)	12/7	12/2	14/4	11/4
Age (years)	54.4 ± 10.6	56.6 ± 9.2	61.2 ± 5.0	62.5 ± 6.3
Axial length (mm)	28.0 ± 1.6	28.7 ± 1.8	23.3 ± 0.7	23.0 ± 1.2
CTGF (pg/ml)	247.6 ± 89.6	182.8 ± 50.2	172.7 ± 48.2	167.7 ± 48.5
HGF (ng/ml)	16.3 ± 7.3	17.8 ± 12.9	12.2 ± 5.5	18.7 ± 4.8

Group A (high myopia with macular hole), Group B (high myopia with macular retinoschisis), Group C (idiopathic macular hole), and Group D (idiopathic epiretinal membrane)

### Vitreal levels of CTGF and HGF

**Fig. 1:**
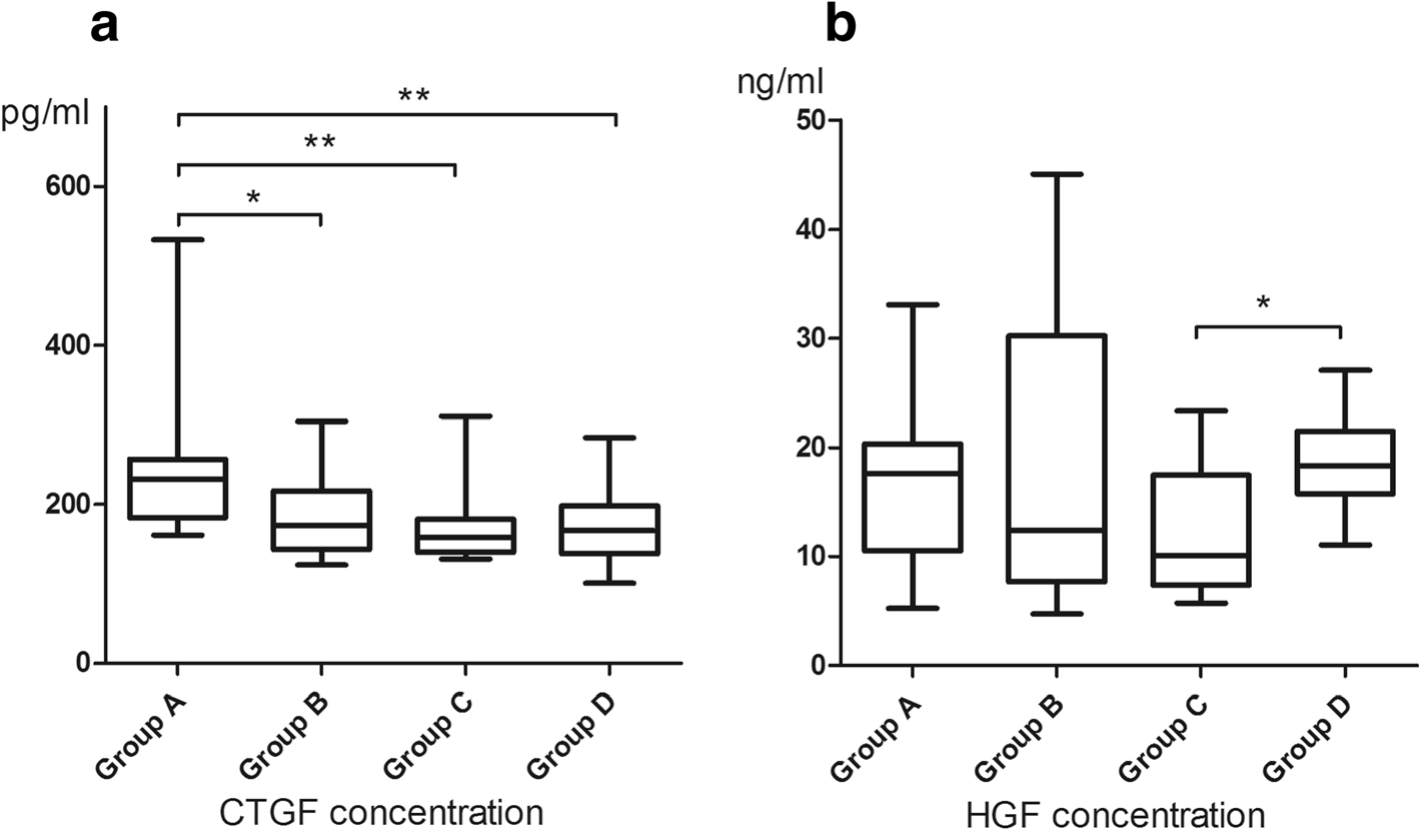
Comparative analysis of vitreal cytokine levels in four subgroups. **a.** The vitreal level of CTGF; **b.** The vitreal level of HGF. (* *P* < 0.05, ** *P* < 0.01)

The vitreal level of CTGF was significantly higher in the high myopia group (220.1 ± 81.3 pg/ml) than in the VMID group (170.4 ± 47.7 pg/ml, *P* < 0.01); in the subgroup analysis, vitreal CTGF was significantly higher in group A than in the other three subgroups (Fig. 1a). There was no significant difference in the vitreal level of HGF between the high myopia (17.0 ± 9.9 ng/ml) and VMID (15.2 ± 6.1 ng/ml) groups. However, the subgroup analysis showed a significantly higher HGF level in group D than in group C (*P* < 0.05) (Fig. 1b).

In the correlation analyses, the CTGF level showed no correlation with age but a significant positive correlation with axial length (Spearman’s rho = 0.41, *P* < 0.01). The level of HGF was not significantly correlated with age or axial length.

## Discussion

Myopia is a highly prevalent eye disease, and its pathogenesis is a focus of ophthalmology research. Previous studies have shown that TGF-β and MMPs play important roles in the pathogenesis of myopia [6–9]. Alterations in TGF-β2 expression is associated with scleral remodeling in myopia development [14]. MMP-2 upregulation was shown to precede myopia development [15], whereas reduced expression of the tissue inhibitor of metalloproteinase was associated with myopia development [16]. But, the associations of CTGF and HGF with myopia are still unclear.

In this study, we explored the expression levels of CTGF and HGF in the vitreous humor of highly myopic eyes. Patients with high myopia complicated with macular hole or macular retinoschisis were included in this study because vitreous specimens could be collected from these patients during vitrectomy. Importantly, we had to exclude certain conditions that potentially disrupt the blood–retinal barrier, such as choroidal neovascularization, proliferative vitreoretinopathy, and any history of laser treatment or ocular surgery. Patients with idiopathic macular hole or idiopathic epiretinal membrane were selected as the control group because these VMIDs involve relatively few factors that interfere with components of the vitreous. Furthermore, vitreous specimens are easily obtained from patients with idiopathic macular hole and idiopathic epiretinal membrane, which are two of the most commonly diagnosed VMIDs. None of these eyes with VMIDs had high myopia. This study confirmed that the axial length was significantly shorter in the VMID than in the high myopia group.

CTGF is a key player in the regulation of extracellular matrix remodeling, fibrosis, and angiogenesis. CTGF is widely expressed in various ocular tissues such as the cornea, trabecular, choroid, and sclera [17]. Previous studies have found that CTGF is associated with corneal wound healing, proliferative diabetic retinopathy, and choroidal neovascularization [18–20]. This study showed, for the first time, that the CTGF level was increased in the vitreous humor of eyes with high myopia, especially in eyes with high myopia complicated with macular hole. This finding suggests that the cytokine CTGF is likely involved in remodeling of the scleral extracellular matrix in myopia and in the pathogenesis of myopic retinopathy. Regarding the potential underlying mechanisms, CTGF expression is regulated by TGF-β [21] and has been correlated with MMP levels in some disease models [22–24]. CTGF regulates matrix production via the ERK (p42/p44 MAPK) and p38 MAPK signaling pathways in certain scenarios [25]. The specific role and the mechanism of CTGF in myopia development requires further study.

HGF was first recognized as a factor that stimulates the proliferation of hepatocytes. It was found soon thereafter that HGF also acts on epithelial cells, vascular endothelial cells, and fibroblasts. HGF has multiple functions in the regulation of wound healing, and angiogenesis [26, 27]. In the present study, the HGF level in the vitreous was measured, for the first time, in highly myopic eyes, but no significant difference was found compared with those without high myopia.

Subgroup analysis unexpectedly showed that the vitreal HGF level was significantly higher in eyes with idiopathic epiretinal membrane than in those with idiopathic macular hole. Iannetti et al. reported elevated levels of TGF-β2 and nerve growth factor in the vitreous humor of patients with idiopathic epiretinal membrane [28]. Zandi et al. revealed differences in vitreal cytokine profiles between eyes with epiretinal membrane and those with macular hole [29]. Our findings also suggested that vitreal cytokine expression differs between epiretinal membrane and macular hole. HGF may be produced in the eye by retinal cells such as Müller cells [26], and it has been demonstrated to induce proliferation and migration of retinal pigment epithelial (RPE) cells [30, 31]. Previous studies found that HGF is associated with proliferative diabetic retinopathy and proliferative vitreoretinopathy [32, 33]. Similarly, HGF-induced Müller cell activation and RPE cell migration may contribute to the formation of epiretinal membrane. Thus, we speculate that HGF is involved in the pathogenesis of idiopathic epiretinal membrane.

## Conclusion

The CTGF level in the vitreous differs between eyes with and without high myopia, and increased CTGF expression may be related to the pathogenesis of high myopia. In addition, increased expression of HGF may be involved in the development of idiopathic epiretinal membrane.

## Abbreviations

CTGF: connective tissue growth factor
HGF: hepatocyte growth factor
VMID: vitreomacular interface disease
TGF: transforming growth factor
MMP: matrix metalloproteinase
